# Loop-mediated Isothermal Amplification and nested PCR of the Internal Transcribed Spacer (ITS) for *Histoplasma capsulatum* detection

**DOI:** 10.1371/journal.pntd.0007692

**Published:** 2019-08-26

**Authors:** Matheus da Silva Zatti, Thales Domingos Arantes, José Alex Lourenço Fernandes, Mônica Baumgardt Bay, Eveline Pipolo Milan, Georggia Fatima Silva Naliato, Raquel Cordeiro Theodoro

**Affiliations:** 1 Institute of Tropical Medicine of Rio Grande do Norte, Federal University of Rio Grande do Norte, Natal, Brazil; 2 Department of Infectious Diseases, Federal University of Rio Grande do Norte, Natal, Brazil; Yale University School of Medicine, UNITED STATES

## Abstract

**Background:**

Histoplasmosis is a neglected disease that affects mainly immunocompromised patients, presenting a progressive dissemination pattern and a high mortality rate, mainly due to delayed diagnosis, caused by slow fungal growth in culture. Therefore, a fast, suitable and cost-effective assay is required for the diagnosis of histoplasmosis in resource-limited laboratories. This study aimed to develop and evaluate two new molecular approaches for a more cost-effective diagnosis of histoplasmosis.

**Methodology:**

Seeking a fast, suitable, sensitive, specific and low-cost molecular detection technique, we developed a new Loop-mediated Isothermal Amplification (LAMP) assay and nested PCR, both targeting the Internal Transcribed Spacer (ITS) multicopy region of *Histoplasma capsulatum*. The sensitivity was evaluated using 26 bone marrow and 1 whole blood specimens from patients suspected to have histoplasmosis and 5 whole blood samples from healthy subjects. All specimens were evaluated in culture, as a reference standard test, and *Hcp100* nPCR, as a molecular reference test. A heparin-containing whole blood sample from a heathy subject was spiked with *H*. *capsulatum* cells and directly assayed with no previous DNA extraction.

**Results:**

Both assays were able to detect down to 1 fg/μL of *H*. *capsulatum* DNA, and ITS LAMP results could also be revealed to the naked-eye by adding SYBR green to the reaction tube. In addition, both assays were able to detect all clades of *Histoplasma capsulatum* cryptic species complex. No cross-reaction with other fungal pathogens was presented. In comparison with *Hcp100* nPCR, both assays reached 83% sensitivity and 92% specificity. Furthermore, ITS LAMP assay showed no need for DNA extraction, since it could be directly applied to crude whole blood specimens, with a limit of detection of 10 yeasts/μL.

**Conclusion:**

ITS LAMP and nPCR assays have the potential to be used in conjunction with culture for early diagnosis of progressive disseminated histoplasmosis, allowing earlier, appropriate treatment of the patient. The possibility of applying ITS LAMP, as a direct assay, with no DNA extraction and purification steps, makes it suitable for resource-limited laboratories. However, more studies are necessary to validate ITS LAMP and nPCR as direct assay in other types of clinical specimens.

## Author summary

Histoplasmosis is a worldwide neglected disease with a high mortality rate associated with HIV/AIDS patients, killing more than tuberculosis in some endemic countries in Latin America. Part of this elevated mortality rate is due to delayed diagnosis and treatment. Here we present two novel methods, one based on Loop-mediated Isothermal Amplification (LAMP) and another on nested Polymerase Chain Reaction (nPCR), for fast, sensitive and specific diagnosis of histoplasmosis. Tests of blood samples spiked with *Histoplasma capsulatum* suggest the possibility of direct application of the LAMP assay proposed herein to clinical specimens without the need for previous DNA extraction and with the added advantage of naked-eye evaluation of the reaction results. Once the assay has been validated in different clinical specimens, it may be a promising tool for fast histoplasmosis screening.

## Introduction

Histoplasmosis, a systemic mycosis distributed worldwide, is caused by *Histoplasma capsulatum sensu lato*, a species complex of pathogenic fungi, which includes at least eight clades distributed in Australian, the Netherlands, Eurasian, North American, Latin American and Africa, according to Kasuga *et al* (2003) [1]. Recently, six clades were added to this species complex, two from Central America, three from Latin America and one clade associated with the long-migratory bat species *Tadarida brasiliensis* and *Mormoops megalophylla* [2].

In the environment, *H*. *capsulatum* occurs saprobiotically in soils enriched with nitrogen compounds from bat guanos and bird feces [3,4]. In humans, infection occurs by inhalation of microconidia suspended in the air. Many outbreaks have been reported due to cave visits, soil survey or basement cleaning [3,5–9]. Immunocompetent individuals only develop a mild or asymptomatic form of the disease. On the other hand, immunocompromised patients may develop progressive disseminated histoplasmosis (PDH), which usually affects brain, bone marrow, lymph nodes and liver [10].

Despite the remarkable phylogenetic diversity of *H*. *capsulatum*, no clinical difference among the different cryptic species has been reported so far. Furthermore, the symptoms of disseminated histoplasmosis are nonspecific, such as fever, cough, weight loss, diarrhea, adynamia, hepatosplenomegaly, hypotension, chills, skin rashes and, in severe cases, altered mental status and respiratory failure [11,12]. Since other systemic diseases such as tuberculosis and other mycoses may also present some of these symptoms, clinical diagnosis is inconclusive [13–16].

The gold standard diagnosis of histoplasmosis is the isolation of its etiological agent in culture medium, followed by microscopic characterization of the mold and its thermal dimorphism. However, these methodologies may require many weeks or months for a conclusive diagnosis [17]. On the other hand, serological assays in urine or serum can be a powerful tool for the rapid diagnosis of histoplasmosis. Nevertheless, such assays are not affordable in developing countries. Also, these assays can vary in sensitivity according to sample source and clinical aspects of the disease [18,19] and may present low specificity due to cross-reactivity with *Coccidioides* spp, *Paracoccidioides* spp, *Blastomyces* spp and *Penicillium marneffei* [18,20,21].

In order to ensure both sensitivity and specificity, many Nucleic Acid Amplification Techniques (NAT) have been applied to the diagnosis of histoplasmosis, such as conventional PCR [22,23], nested PCR (nPCR) [24–26] and real time quantitative PCR (qPCR) [27–29]. Nevertheless, all NATs require specific equipment, well-trained experts and are not suitable for resource-limited laboratories. For these reasons, Isothermal Nucleic Acid Amplification Techniques (INAT) are more suitable alternatives for molecular diagnosis of infectious diseases. Heretofore, there are two INATs applied to the detection of *H*. *capsulatum* in clinical samples: a Loop-mediated Isothermal Amplification (LAMP) targeting the single-copy gene *Hcp100* [30] and a Rolling Circle Amplification (RCA) of the Internal Transcribed Spacer (ITS) region [31]. The *Hcp100* LAMP is specific for *H*. *capsulatum sensu lato*, although the use of a single-copy gene as a target may contribute to some discrepancies observed for detection of the pathogen in urine specimens [30]. Although the RCA technique targets the Internal Transcribed Spacer (ITS) which is a multicopy region, this method requires a previous PCR step for isolation of the target to be recognized by the padlock probes, which makes this technique as limiting as conventional NATs.

LAMP is a highly efficient, sensitive, specific and cost-effective isothermal amplification method that uses at least four primers, recognizing six different regions in the target sequence and results in a self-primed DNA. [32]. Forward Inner Primer (FIP) and Backward Inner Primer (BIP) have inverted sequences attached at the 5’ end, named F1c and B1c, which are complementary to an internal sequence from the amplified strand, forming a loop at each extremity of a single strand DNA. The outer primers (F3 and B3) anneal upstream to the FIP and BIP, acting as a binding site for DNA polymerase, which, in the LAMP reaction, also contains the strand displacement activity. In addition, LAMP results can be observed using several strategies with minimal ambiguity, including real-time turbidimetry (magnesium pyrophosphate formation) [33], fluorescent compounds (Sybr Green, Eva Green, SYTO, calcein) [34], magnesium colorimetric titration (hydroxynaphtol blue) [35], fluorescent-labeled probes/quencher-labeled primers [36], dye-labeled primers [37] and pH-sensitive dyes [38].

In this work, new LAMP primers were developed for *H*. *capsulatum sensu lato* detection in clinical samples. To increase the reaction sensitivity, the ITS region was used as target, since it is a multicopy sequence and also because it is considered an important barcoding sequence for fungal identification, being conserved among *H*. *capsulatum* strains and divergent from other fungi, ensuring high specificity [39–44]. In order to compare the efficiency of LAMP and NAT, we also designed two primer sets for nPCR targeting the same genomic region (ITS) of *H*. *capsulatum*. Our results indicate that LAMP targeting the ITS region is a powerful and reliable high-sensitivity tool for the diagnosis of histoplasmosis, mainly in resource-limited laboratories.

## Methods

### Ethics statement

The study protocol was approved by the Ethics Committee of Federal University of Rio Grande do Norte under protocol number CAAE:39640614.8.0000.5537. All specimens were collected for the routine diagnosis of subjects admitted at Giselda Trigueiro Hospital during this study. Written consent was obtained from all patients and healthy individuals. All samples were anonymized for use in this study.

### Clinical specimens: Collection, culture and DNA extraction

Twenty-six bone marrow and one whole blood specimens were prospectively and consecutively obtained from HIV/AIDS patients, with symptoms of PDH, admitted at Giselda Trigueiro Hospital in Natal, Rio Grande do Norte state, Brazil, from July 2015 to July 2018. Additionally, 5 whole blood specimens were obtained from healthy subjects. Symptoms of PDH include: fever (≥ 38°C) and one or more of those following signs: adenomegaly, hepatosplenomegaly, pulmonary infiltrate, pancytopenia and splenic microabscesses. Samples were drawn at admission for *Histoplasma* culture on Sabouraud Dextrose Agar medium (SDA) with chloramphenicol (50 mg/L) and incubated at 25°C. All specimens were collected and immediately cultured before being transferred to EDTA tubes and sent to the Mycology Laboratory of the Institute of Tropical Medicine of RN, Brazil, where 100 μL of the sample was again cultivated on SDA medium with chloramphenicol (50 mg/L), under sterile conditions, and incubated at 25°C. The remaining specimen was stored at -80°C, until DNA extraction. Culture was maintained for 120 days or until fungal growth was observed. The diagnosis of histoplasmosis was confirmed only after morphological visualization of fungal micro and macro conidia.

The DNA was extracted from clinical specimens using 100 μL of the sample with the DNeasy Blood & Tissue kit (Qiagen, Inc., Hilden, Germany) according to the manufacturer's instructions. Briefly, the sample was incubated with 20 μL of proteinase K (100 mg/ml) and 200 μL Buffer AL at 56°C for 10 min. The lysate was precipitated with ethanol P.A. and placed into a DNeasy Mini Spin column, followed by two wash steps. DNA was eluted in nuclease-free water and stored at -20°C. All samples were renamed before molecular assays for blind evaluation to avoid confirmatory bias. Standards for Reporting of Diagnostic Accuracy (STARD) flow chart of subjects and checklist are provided in S1 Fig and S1 Checklist, respectively.

### DNA extraction from fungal cultures

DNA was extracted from *H*. *capsulatum* strains isolated in this work and from negative fungal controls (Table 1), according to McCullough *et al* (2000) [45], with some modifications as follows: after 3–5 days of fungal growth on SDA medium, about 2 to 3 mold fragments, 1 cm^2^ each, were transferred to a sterile mortar and frozen with liquid nitrogen. The material was triturated and transferred to a 1.5 ml tube containing 500 μL of lysis buffer (50 mM Tris HCl, 50 mM EDTA, 2% SDS) and then incubated at 65°C for 60 min. After incubation, 500 μL of 5M potassium acetate were added to the mixture and the lysate was maintained at -20°C overnight. The material was centrifuged at 13,000 x g for 10 min and the supernatant transferred to a new 1.5 ml tube containing 600 μL of cold ethanol P.A. After centrifugation at 13,000 x g for 10 min, the supernatant was discarded, and the DNA was incubated at 65°C for 60 min to dry. Finally, DNA was solubilized in 60 μL of nuclease-free water and stored at -20°C.

**Table 1:** Fungi used as specificity controls in ITS LAMP and nPCR assays.

Fungal species	DNA amount	PCR *panfungal*	ITS LAMP	ITS nPCR
*Aspergillus niger*	20 ng	+	-	-
*Aspergillus flavus*	20 ng	+	-	-
*Candida* spp.	20 ng	+	-	-
*Cladophialophora carrionii*	20 ng	+	-	-
*Cryptococcus gattii*	20 ng	+	-	-
*Cryptococcus neoformans*	20 ng	+	-	-
*Histoplasma capsulatum*	20 ng	+	+	+
*Microsporum canis*	20 ng	+	-	-
*Paracoccidioides brasiliensis*	20 ng	+	-	-
*Sporothrix brasiliensis*	20 ng	+	-	-
*Trichophyton mentagrophytes*	20 ng	+	-	-
*Trichosporon* spp.	20 ng	+	-	-

### LAMP primer set design and reaction

**Fig 1:**
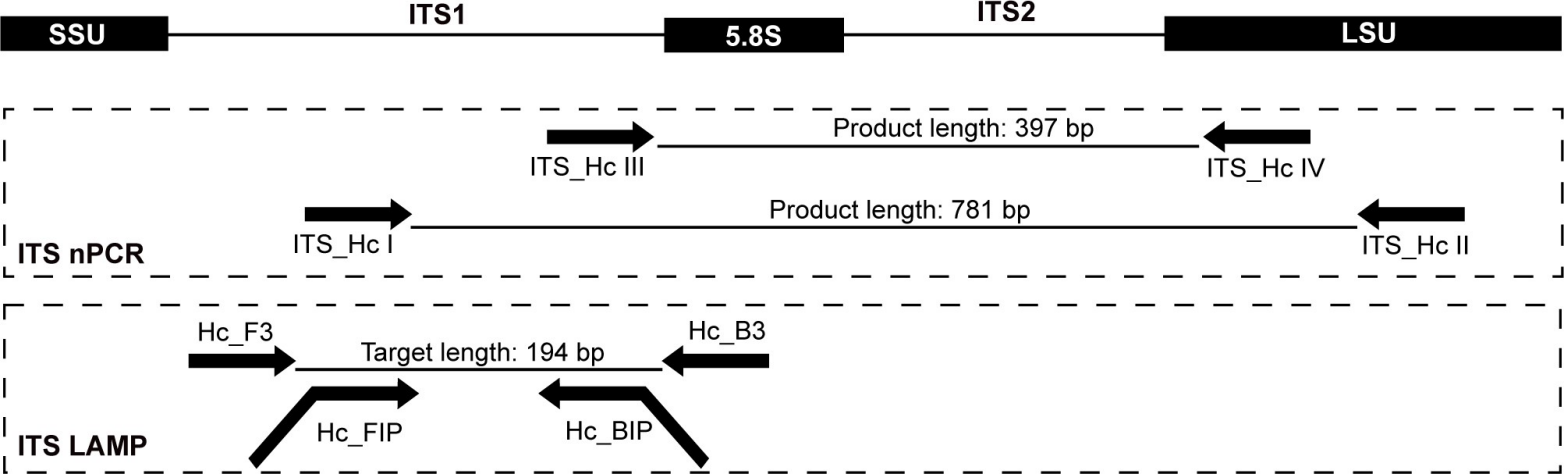
Illustrative diagram showing the hybridization regions of ITS LAMP and nPCR primer sets. SSU, small ribosomal subunit (18S); ITS, Internal Transcribed Spacer; LSU, large ribosomal subunit (28S).

Ninety-eight *H*. *capsulatum* ribosomal RNA (18S - ITS1–5.8S - ITS2 - 28S) sequences were obtained from the GenBank (see Accession number section) database and aligned by ClustalW, available in MEGA (version 7.0) [46–48]. The consensus sequence was generated in BioEdit (version 7.0.2) considering 95% identity among all sequences. The LAMP primer set was manually designed within conserved regions of ITS1 (Table 2 and Fig 1) according to Notomi *et al*. (2000) [32] and following the guidelines of the "A Guide to LAMP Primer Designing (Primer Explorer V4)" (Eiken Chemical Co., Ltd., Tokyo, Japan) (https://primerexplorer.jp/e/v4_manual/index.html).

**Table 2:** Primers used in LAMP and nPCR assays for *H*. *capsulatum* detection.

Primer	Sequence (position^‡^)	Reference
**Hc_F3**	TACCCGGCCACCCTTGTC (62–79)	This work
**Hc_FIP**	GGAIAAGITCCCCCGGCAGTACCGGACCTGTTGCITCG (80–134)	
**Hc_B3**	ATGTCGGAACCAAGAGATCC (236–255)	
**Hc_BIP**	CCGTCGGTGAAYGATTGGCGGTTGTTGAAAGTTTTGACTGGA (171–235)	
**ITS_HcI**	TGTCTACCGGACCTGTTGC (76–94)	This work
**ITS_HcII**	CCACCCATTTGGAGCTGCA (839–857)	
**ITS_HcIII**	AGAGCGATAATAATCCAGTC (201–220)	
**ITS_HcIV**	GATATGCTTAAGTTCAGCG (580–598)	
**Hc I**	GCGTTCCGAGCCTTCCACCTCAAC	Reference [24]
**Hc II**	ATGTCCCATCGGGCGCCGTGTAGT	
**Hc III**	GAGATCTAGTCGCGGCCAGGTTCA	
**Hc IV**	AGGAGAGAACTGTATCGGTGGCTTG	

^**‡**^ according the accession number AF038353.1

LAMP reaction was carried out in a final volume of 12.5 μL, containing 1x *Bsm* reaction buffer [20 mM Tris-HCl (pH 8.8), 10 mM KCl, 10 mM (NH_4_)_2_SO_4_, 2 mM MgSO_4_, 0.1% (v/v) Tween 20], 4 U of *Bsm* polymerase (Thermo Fisher Scientific, Inc., Massachusetts, EUA), 1.6 μM of each inner primer (Hc_FIP and Hc_BIP), 0.4 μM of each outer primer (Hc_F3 and Hc_B3), 2.8 mM of each dNTP, 0.6 M of betaine, 10 mM of magnesium sulfate and 2 μL of DNA. The reaction mixture was heated at 95°C for 2 min, followed by cooling on ice, prior to the addition of the *Bsm* polymerase. The reaction was incubated at 60°C for 120 min. *Bsm* polymerase was inactivated at 80°C for 10 min. LAMP products were observed after electrophoresis on 1.5% agarose gel stained with ethidium bromide or by addition of 1 μL of 10-fold diluted Sybr Green I dye (Lonza, Inc., Basel, Switzerland) to the reactions. For positive amplification reactions the dye color changes from orange to green under white or UV light.

### ITS nPCR primer set design and reaction

As for LAMP, the ITS nPCR primer set was designed within conserved regions, specific for *H*. *capsulatum sensu lato*. The outer primers (ITS_HcI and ITS_HcII) flank 781 bp of 5.8S-ITS2 and the inner primers (ITS_HcIII and ITS_HcIV) amplify a 397 bp fragment (Table 2 and Fig 1).

The first reaction was carried out in a final volume of 12.5 μL, containing 1x HF buffer (containing 1.5 mM MgCl_2_), 100 μM of each dNTP, 0.1 μM of each outer primer (ITS_HcI and ITS_HcII), 3% DMSO, 0.25 U of Phusion High-Fidelity DNA polymerase (New England BioLabs, Inc., Massachusetts, USA) and 2 μL of DNA. Thermal cycling was performed in a Veriti 96-well thermal cycler (Applied Biosystems, Inc, California, USA), as follows: 98°C for 30 sec; 40 cycles of 98°C for 10 sec, 64°C for 30 sec, 72°C for 30 sec; and a final extension at 72°C for 5 min. The second reaction mix was identical to the first, except for use of 0.2 μM of each inner primer (ITS_HcIII and ITS_HcIV), 50 μM of each dNTP, and 1 μL of the first reaction product as template. The thermal cycling conditions were: 98°C for 30 sec; 30 cycles of 98°C for 10 sec, 60°C for 30 sec, 72°C for 20 sec; and a final extension at 72°C for 5 min. Amplicons were observed after electrophoresis on 1.5% agarose gel stained with ethidium bromide.

### *Hcp100* nPCR reaction

In order to ensure the presence of *Histoplasma* DNA in clinical specimens, *Hcp100* nPCR [24,25] was carried out with the modifications suggested by Taylor *et al*. (2005) [5]. The first reaction was performed in a final volume of 12.5 μL, containing 1x CG buffer (containing 1.5 mM MgCl_2_), 200 μM of each dNTP, 0.2 μM of each outer primer (Hc I and Hc II), 0.25 U of Phusion High-Fidelity DNA polymerase (New England BioLabs, Inc., Massachusetts, USA) and 2 μL of DNA. The thermal cycling conditions were: 98°C for 30 sec; 40 cycles of 98°C for 10 sec, 50°C for 30 sec, 72°C for 30 sec; and a final extension at 72°C for 5 min. The second reaction was identical to the first, except for use of 0.2 μM of each inner primer (Hc III and Hc IV), 50 μM of each dNTP and 1 μL of the first reaction product as template. The thermal cycling conditions were: 98°C for 30 sec; 40 cycles of 98°C for 10 sec, 68°C for 30 sec, 72°C for 30 sec; and a final extension at 72°C for 5 min. Amplicons were observed after electrophoresis on 1.5% agarose gel stained with ethidium bromide.

### GAPDH PCR reaction

To ensure that DNA extracted from clinical specimens was intact and amplifiable, the GAPDH (glyceraldehyde 3-phosphate dehydrogenase) gene was used as a housekeeping reference control. The reaction was performed in a final volume of 12.5 μL, containing 1x GoTaq Reaction Buffer (containing 1.5 mM MgCl_2_), 200 μM of each dNTP, 0.2 μM of forward GAPDH primer (5’–CAAGGTCATCCATGACAACTTTG–3’) and reverse GAPDH primer (5’–GTCCACCACCCTGTTGCTGTAG-3’) from the RevertAid H Minus First Strand cDNA Synthesis Kit (Thermo Scientific), 0.25 U of GoTaq DNA polymerase (Promega, Co., Wisconsin, USA) and 2 μL of DNA. The thermal cycling conditions were: 95°C for 3 min; 35 cycles of 95°C for 30 sec, 58°C for 30 sec, 72°C for 45 sec; and a final extension at 72°C for 10 min.

### Limit of detection and reproducibility

To determine the limit of detection (LOD) of both ITS LAMP and nPCR, *H*. *capsulatum* DNA was diluted 10-fold (from 10 ng/μL to 0.1 fg/μL) and assayed. Four to six technical replicates were performed for dilutions from 10 pg/μL to 0.1 fg/μL in order to determine the assays reproducibility. DNA concentration was determined using the fluorometer Qubit 2.0 (Thermo Fisher Scientific, Inc., Massachusetts, USA) according to the manufacturer's instructions. Considering the genome length of *H*. *capsulatum* is 33.03 Mb (accession number: GCA_000149585.1) and one base pair equals 660 g/mol, the number of detected genomes of *H*. *capsulatum* was determined by using the following formula: Genome copies = (amount of DNA x 6.022 x 10^23^) / (genome length x 660 x 10^9^).

### Specificity assay

ITS nPCR and LAMP assays were conducted with 20 ng of DNA per reaction from clinical isolates and from reference strains of pathogenic fungi. All controls were also PCR-amplified with the *panfungal* primers ITS4 (5'-TCCTCCGCTTATTGATATGC-3') and ITS5 (5'-GGAAGTAAAAGTCGTAACAA-3'), described by White *et al*. (1990) [49] to ensure DNA quality. Table 1 shows the fungal species used as specificity controls for ITS LAMP and nPCR.

### Direct ITS LAMP and ITS nPCR assays

To perform the ITS LAMP and nPCR as direct assays, yeast cells of *H*. *capsulatum* were serially diluted from 10^4^ yeasts/μL to 1 yeast/μL in either PBS or heparin-containing whole blood from a healthy control. Stored bone marrow samples were not suitable for this assay, since freezing ruptures the cellular membranes. Yeasts in PBS were heat-treated at 100°C for 10 minutes before being assayed. One microliter of each dilution was directly assayed in ITS LAMP and nPCR without any previous DNA extraction or purification steps.

### Statistical analysis

McNemar’s test exact *p-value* was used to evaluate the difference in sensitivity between diagnostic methods. *P-value >* 0.05 was assumed as not statistically significant. The *kappa* statistic was calculated to evaluate the agreement between the reference and herein proposed methods for *H*. *capsulatum* detection in clinical samples [50]. The interpretation for *kappa* values was as follow: 0.00–0.20, poor agreement; 0.21–0.40, fair agreement; 0.41–0.60, moderate agreement; 0.61–0.80, good agreement; 0.81–1.00, excellent agreement [50]. Clinical sensitivity, specificity and predictive values were inferred using culture or *Hcp100* nPCR as reference.

### Accession numbers

KF443065.1; JQ218359.1; JQ218358.1; JQ218357.1; JQ218356.1; JQ218355.1; JQ218354.1; JQ218353.1; JQ218352.1; JQ218351.1; JQ218350.1; JQ218349.1; JQ218348.1; JQ218347.1; JQ218345.1; JQ218344.1; JQ218343.1; JQ218342.1; JQ218341.1; JQ218340.1; JQ218339.1; JQ218338.1; JQ218337.1; JQ218336.1; JQ218335.1; HM439693.1; FJ011535.1; KC693555.1; KC693554.1; KC693553.1; KC693552.1; KC693551.1; KC693550.1; KC693549.1; KC693548.1; KC693547.1; KC693546.1; KC693545.1; KC693544.1; KC693540.1; KC693541.1; KC693542.1; KC693543.1; KC693539.1; KC693538.1; KC693537.1; KC693536.1; KC693535.1; KC693530.1; KC693531.1; KC693532.1; KC693533.1; KC693534.1; KC693529.1; KC693528.1; KC693527.1; KC693526.1; KC693525.1; KC693524.1; KC693523.1; KC693522.1; KC693521.1; KC693520.1; KC693519.1; KC693518.1; KC693517.1; KC693516.1; KC693515.1; KC693514.1; KC693513.1; KC693512.1; KC693511.1; KC693510.1; KC693507.1; KF724850.1; KF724849.1; KF724848.1; KF724847.1; KF724846.1; KF724845.1; KF724844.1; KF724843.1; KF724842.1; AF322387.1; AF322386.1; AB071831.1; AB071828.1; AF322385.1; AF322384.1; AF322383.1; AF322382.1; AF322381.1; AF322380.1; AF322379.1; AF322378.1; AF322377.1; AF038354.1; AF038353.1.

## Results

### LAMP primer set design

According to the alignment of 98 sequences of rDNA from *H*. *capsulatum*, the ITS 1 region was shown to be the most conserved for design of the LAMP primer set. However, due to some polymorphisms in this region, three inosines were inserted in the primer Hc_FIP and one degenerate base in the primer Hc_BIP (Table 2 and S2 Fig).

### Limit of detection and reproducibility

**Fig 2:**
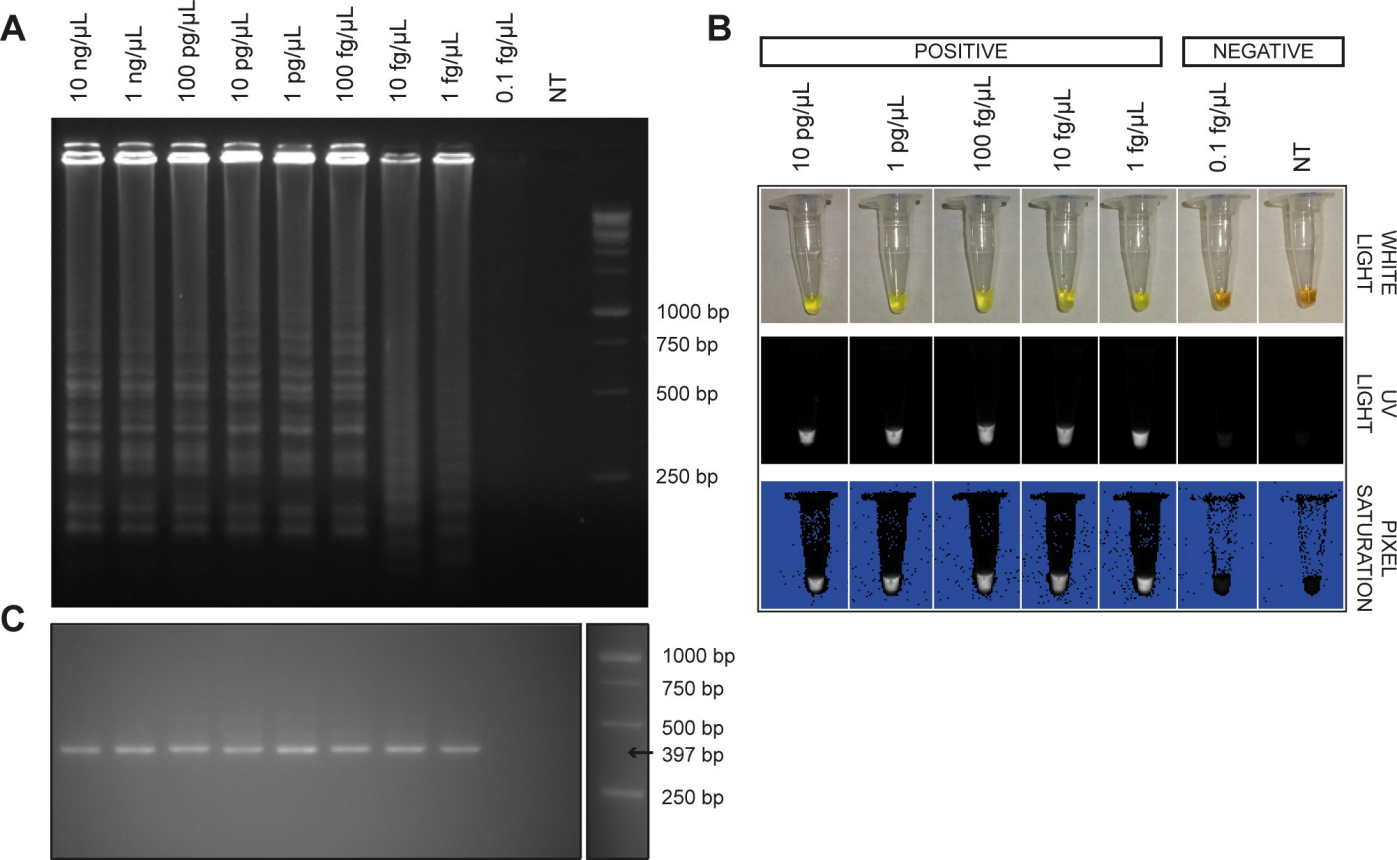
The LOD of ITS LAMP and nPCR for different concentrations (from 10 ng/μL to 0.1 fg/μL) of *H*. *capsulatum* DNA. A: ITS LAMP products in 1.5% agarose gel stained with ethidium bromide; B: ITS LAMP product observed by addition of Sybr Green I in the reaction tube under white light and UV light, respectively; C: ITS nPCR product in 1.5% agarose gel stained with ethidium bromide; NT: No Template control.

As shown in Fig 2, both ITS LAMP and nPCR can detect down to 1 fg/μL of *H*. *capsulatum* DNA. However, reproducible detection only occurs down to 100 fg/μL (Table 3).

**Table 3:** Reproducibility of the Limit of Detection for ITS LAMP and nPCR assays.

Dilution	ITS LAMP	ITS nPCR
1 pg/μL	100% (6/6)	100% (6/6)
100 fg/μL	100% (6/6)	100% (6/6)
10 fg/μL	83% (5/6)	83% (5/6)
1 fg/μL	33% (2/6)	33% (2/6)
0.1 fg/μL	0% (0/4)	0% (0/6)

### Validation and assay specificity

**Fig 3:**
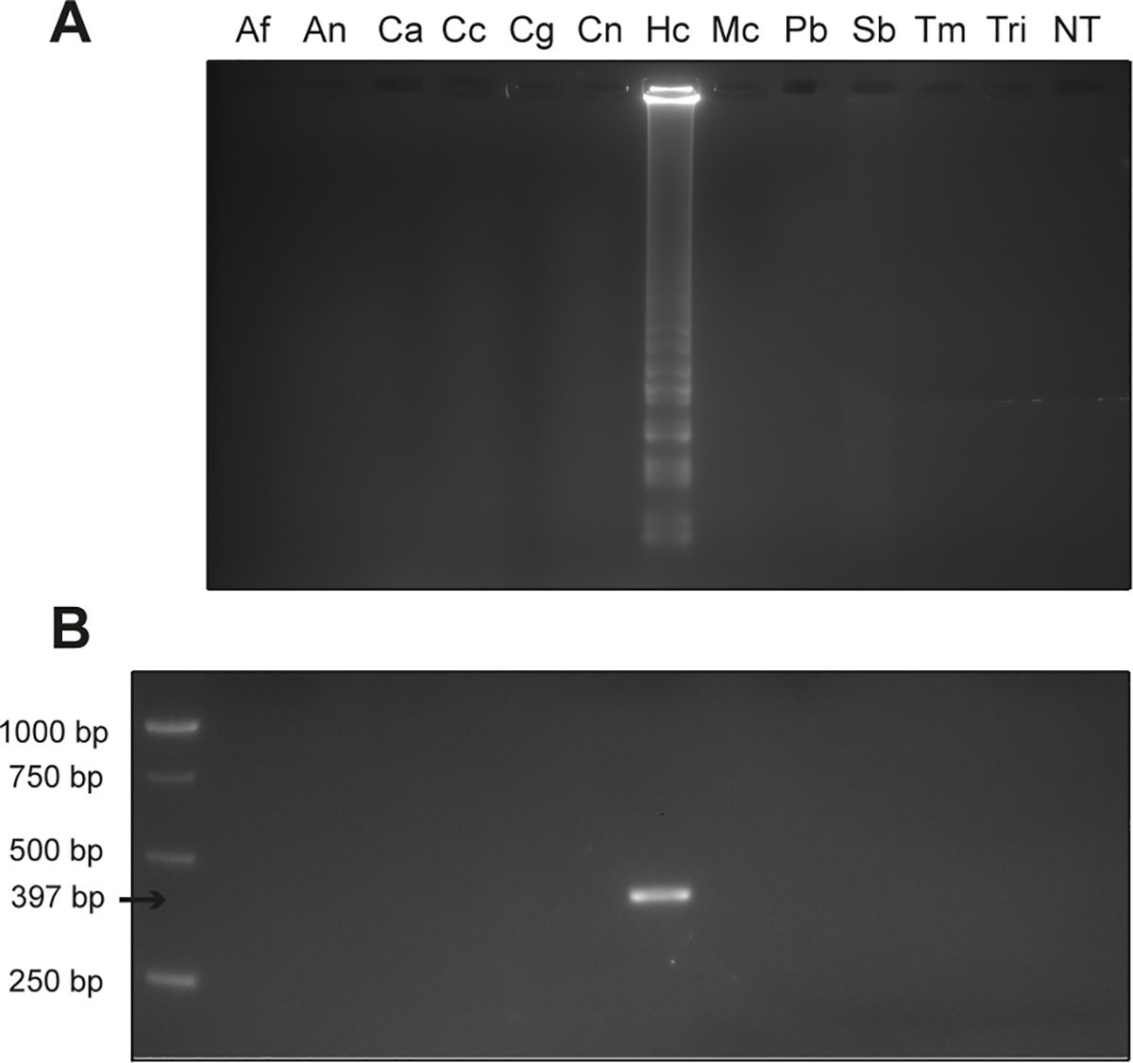
**Specificity assay for ITS LAMP (A) and ITS nPCR (B).** NT, No Template; Af, *Aspergillus flavus*; An, *Aspergillus niger*; Ca, *Candida* spp; Cc, *Cladophialophora carrionii*; Cg, *Cryptococcus gattii*; Cn, *Cryptococcus neoformans*; Hc, *Histoplasma capsulatum*; Mc, *Microsporum canis*; Pb, *Paracoccidioides brasiliensis*; Sb, *Sporothrix brasiliensis*; Tm, *Trichophyton mentagrophytes*; Tri, *Trichosporon* spp.

The ITS LAMP and nPCR showed no cross-reactivity when assayed with DNA from other pathogenic or environmental fungi (Table 1). Therefore, these two methods specifically detect *H*. *capsulatum* DNA (Fig 3). Sequences from some fungi not available in our lab were accessed in the GenBank Data Base and aligned with each primer, ensuring that ITS LAMP and nPCR primers recognize only *H*. *capsulatum* DNA sequence, even when compared to evolutionarily closely-related fungi, such as *P*. *brasiliensis*, *Blastomyces dermatitidis* and *Coccidioides* spp (S3 Fig). Moreover, both methods were able to detect isolates from all geographical clades described by Kasuga *et al*. (2003), including *Histoplasma capsulatum* var. *duboisii*, from the African clade. However, the Eurasian and Australian clades were not available in our laboratory to test (S1 Table).

### Biological specimens

Twenty-six bone marrow and one whole blood specimens were obtained from patients with HIV/AIDS from Giselda Trigueiro Hospital (Table 4). Group A (n = 11) represents culture-positive histoplasmosis patients, whereas those patients who had symptoms of PDH, but negative culture for *Histoplasma*, were allocated to group B (n = 16). Heathy control patients were allocated to group C (n = 5).

**Table 4:** Results of molecular assays and culture of the clinical samples from patients suspected of PDH admitted at Giselda Trigueiro Hospital in Natal, Rio Grande do Norte State, Brazil.

Sample	Group	Sample type	DNA amount	*Hcp100*nPCR	ITS nPCR	ITS LAMP	Fungal culture (strain)	Coinfection
**HGT028**	A	BM	88 ng	-	+	-	*H*. *capsulatum* (HC-2)	HIV, DTB
**HGT039**	A	BM	71 ng	+	-	-	*H*. *capsulatum* (HC-3)	HIV, PTB
**HGT056**	A	BM	100 ng	+	+	+	*H*. *capsulatum* (HC-4)	HIV
**HGT048**	A	BM	100 ng	+	+	+	*H*. *capsulatum* (HC-5)	HIV
**HGT068**	A	BM	100 ng	-	-	-	*H*. *capsulatum* (HC-6)	HIV
**HGT074**	A	BM	100 ng	+	+	+	*H*. *capsulatum* (HC-7)	HIV
**HGT075**	A	BM	56 ng	-	+	+	*H*. *capsulatum* (HC-8)	HIV
**HGT072**	A	BM	100 ng	+	+	+	*H*. *capsulatum* (HC-9)	HIV
**HGT079**	A	BM	111 ng	-	-	-	*H*. *capsulatum* (HC-10)	HIV
**HGT083**	A	BM	88 ng	-	-	-	*H*. *capsulatum* (HC-11)	HIV
**HGT087**	A	WB	128 ng	+	+	+	*H*. *capsulatum* (HC-12)	HIV, DTB
**HGT082**	B	BM	128 ng	-	-	-	*-*	VLS
**HGT032**	B	BM	100 ng	-	-	-	*Cryptococcus* spp.	HIV
**HGT038**	B	BM	66 ng	-	-	-	*Cryptococcus* spp.	HIV
**HGT061**	B	BM	109 ng	-	-	+	-	HIV, TB
**HGT052**	B	BM	107 ng	-	-	-	-	HIV
**HGT054**	B	BM	100 ng	-	-	-	-	HIV
**HGT055**	B	BM	100 ng	-	-	-	-	HIV
**HGT057**	B	BM	100 ng	-	-	-	-	HIV
**HGT058**	B	BM	59 ng	-	-	-	-	HIV
**HGT059**	B	BM	110 ng	-	-	-	-	NA
**HGT062**	B	BM	100 ng	-	-	-	-	HIV, VLS
**HGT063**	B	BM	32 ng	-	-	-	-	NA
**HGT069**	B	BM	74 ng	-	-	-	-	HIV, VLS
**HGT070**	B	BM	100 ng	-	-	-	-	HIV, DTB
**HGT071**	B	BM	54 ng	-	-	-	-	HIV
**HGT073**	B	BM	100 ng	-	-	-	-	HIV
**C1**	C	WB	91 ng	-	-	-	-	NA
**C2**	C	WB	26 ng	-	-	-	-	NA
**C3**	C	WB	120 ng	-	-	-	-	NA
**C4**	C	WB	105 ng	-	-	-	-	NA
**C5**	C	WB	106 ng	-	-	-	-	NA

BM, bone marrow; WB, whole blood; HIV, human immunodeficiency virus; TB, tuberculosis; VLS, visceral leishmaniasis; PTB, pulmonary tuberculosis; DTB, disseminated tuberculosis; NA, not available; +, positive; -, negative.

The median time for growth and morphological identification of *H*. *capsulatum* in culture was 28 ± 34.2 days (median ± standard deviation; ranging from 16 to 119 days). Morphologically-characterized *H*. *capsulatum* isolated on culture was confirmed by *Hcp100* PCR using the primers Hc III and Hc IV as previously described [24,25].

The ITS LAMP and ITS nPCR presented a sensitivity of 54% (6/11) and 64% (7/11) and a specificity of 95% (20/21) and 100%, respectively, when the culture results were used as reference. (Table 5). When the *Hcp100* nPCR was used as reference, both ITS LAMP and nPCR reached 83% (5/6) and 92% (24/26) sensitivity and specificity, respectively (Table 6). The GAPDH gene, used as internal control, was amplified in all clinical specimens (S4 Fig).

**Table 5:** Comparison of sensitivity and specificity parameters of the molecular methods used in this work to detect *H*. *capsulatum* in biological samples using culture as reference.

	ITS nPCR	ITS LAMP
Sensitivity	0.64	0.54
Specificity	1.00	0.96
Positive Predictive Value (PPV)	1.00	0.86
Negative Predictive Value (NPV)	0.85	0.81
*kappa* statistic	0.88	0.82
MacNemar’s test Exact *p-value*	0.13	0.22

**Table 6:** Comparison of sensitivity and specificity parameters of culture, ITS LAMP and nPCR methods for detection of *H*. *capsulatum* in biological samples, using *Hcp100* nPCR as reference.

	ITS nPCR	ITS LAMP
Sensitivity	0.83	0.83
Specificity	0.93	0.93
Positive Predictive Value (PPV)	0.71	0.71
Negative Predictive Value (NPV)	0.96	0.96
*kappa* statistic	0.91	0.91
MacNemar’s test Exact *p-value*	1.00	1.00

### Direct assays

**Fig 4:**
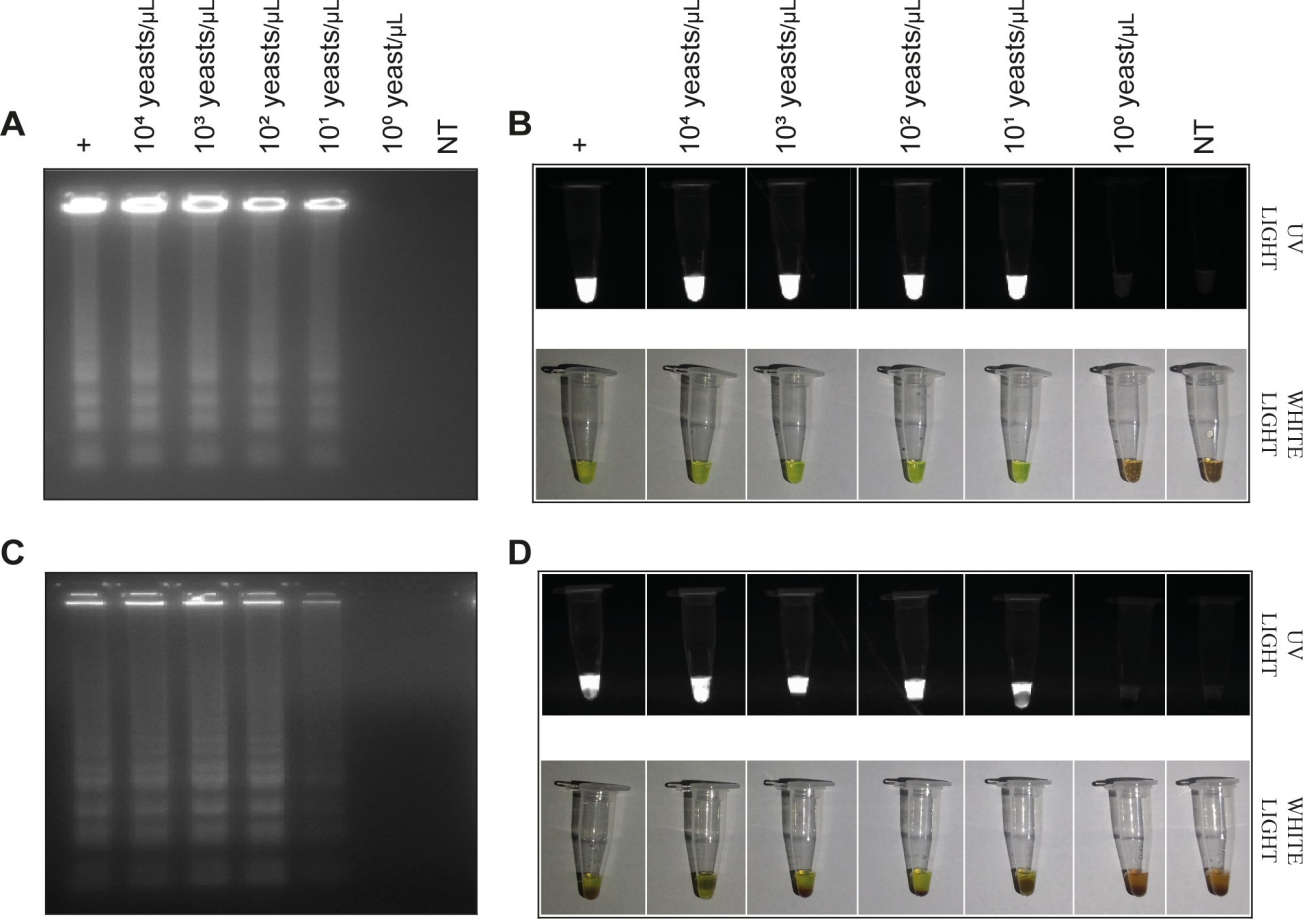
ITS LAMP on crude samples spiked with different concentrations of alive *H*. *capsulatum* yeasts. In A and B, yeasts were diluted in PBS, heat-treated and assayed. In C and D, yeasts were diluted in heparin-containing whole blood and direct assayed. ITS LAMP products were visualized on an agarose gel stained with ethidium bromide (A and C) and by addition of Sybr Green I, under UV and white lights (B and D).

**Fig 5:**
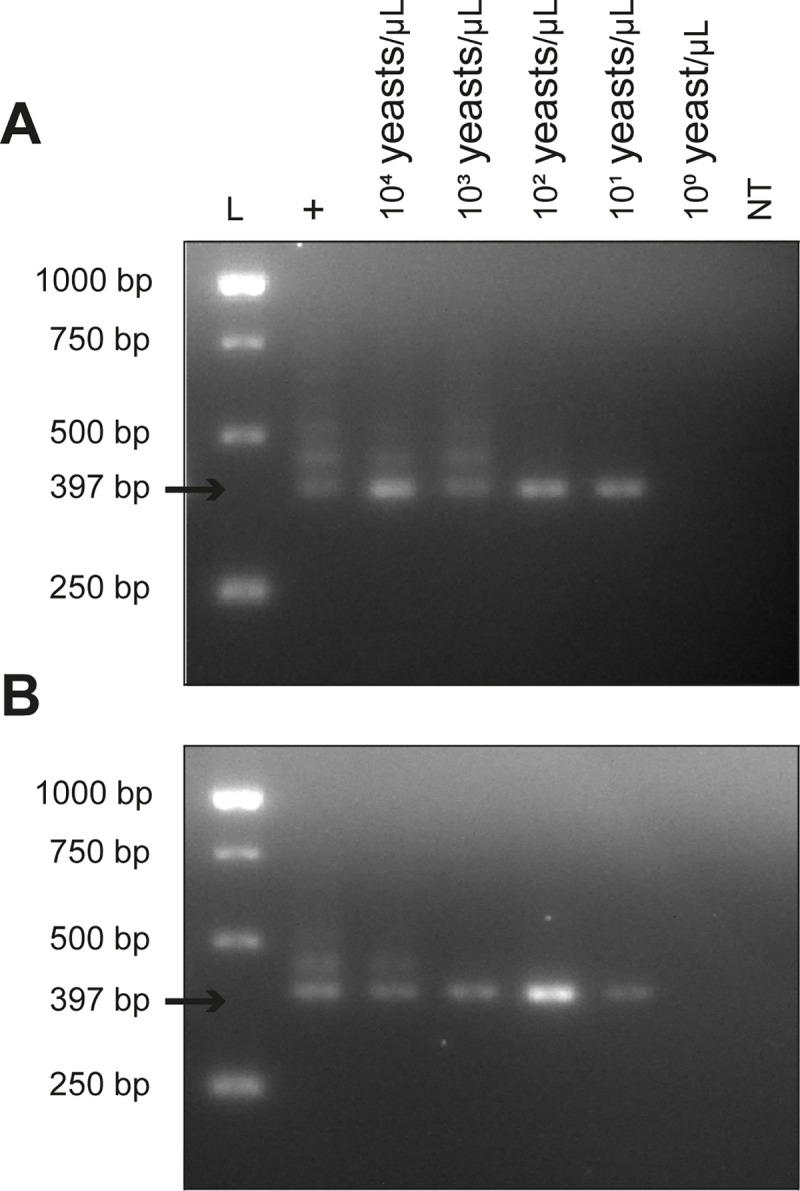
ITS nPCR on crude samples spiked with different concentrations of live *H*. *capsulatum* yeasts. In A, yeasts were diluted in PBS, heat-treated and assayed. In B, yeasts were diluted in heparin-containing whole blood and direct assayed by ITS nPCR. The amplification of a fragment of 397 bp was considered a positive reaction.

To evaluate the performance of ITS LAMP and nPCR as direct assays, without previous DNA extraction, heat-treated PBS and heparin-containing whole blood spiked with *H*. *capsulatum* yeasts were used. Both ITS LAMP and nPCR assays were able to detect as few as 10 yeast cells in either PBS and whole blood samples (Figs 4 and 5). Moreover, LAMP allowed immediate evaluation of the reaction results by addition of SYBR Green I after incubation (Fig 4B and 4D).

### Cost and time consumption

**Fig 6:**
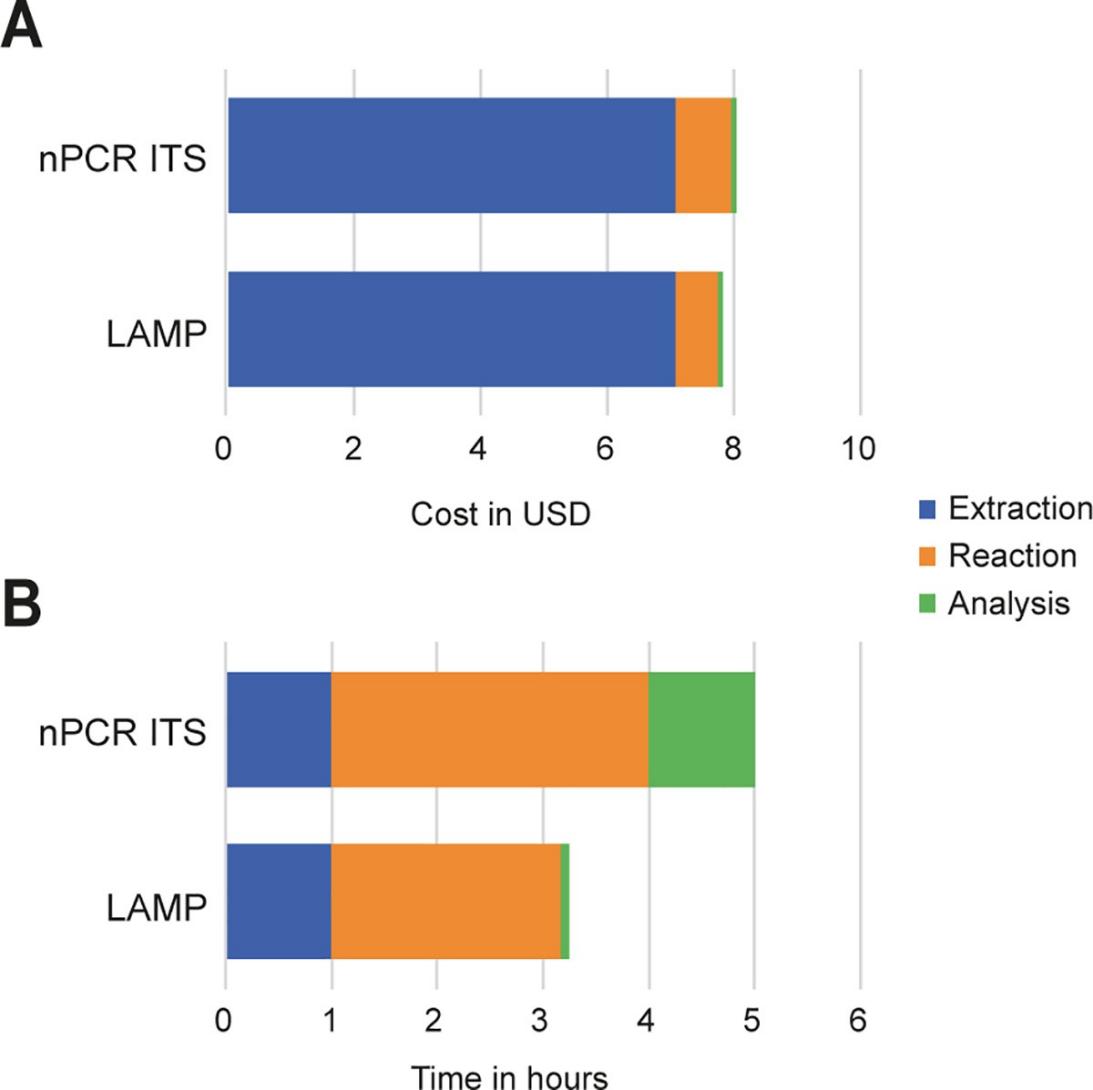
Cost (A) and time (B) consumed for ITS LAMP and nPCR assays.

The cost expended by both techniques was very close, but the ITS LAMP assay was slightly less expensive than nPCR. For the entire procedure, nPCR cost was USD 8.03/sample, whereas LAMP cost was USD 7.82/sample, but with the great added advantage of not requiring a thermocycler or an electrophoresis apparatus (Fig 6). The DNA extraction step represents 90.4% and 88.2% of the LAMP and nPCR total cost, respectively. When LAMP and nPCR were applied as direct assays, without DNA extraction and purification steps, the total cost was USD 0.75 for LAMP and USD 0.95 for nPCR.

When analyzing the time required to perform each technique, LAMP was performed in less than 200 minutes whereas nPCR took at least 300 minutes (Fig 6). These results show that LAMP is faster and less expensive than nPCR to detect *H*. *capsulatum*.

## Discussion

Recent estimates indicate there are approximately 100,000 annual cases of PDH in AIDS patients worldwide, from which 80% result in death [51]. This high mortality rate is mainly due to the lack of adequate treatment, which can only be achieved with a fast and efficient diagnosis. However, clinical symptoms, radiological and routine laboratory tests are nonspecific and histoplasmosis is often misdiagnosed as drug-resistant tuberculosis or pneumocystosis [52–55]. In fact, delayed diagnosis of PDH is an important cause of the high mortality rate in South America [55–57]. Among people living with HIV, histoplasmosis is an important AIDS-related infectious disease in endemic Latin American countries, killing even more than tuberculosis [58].

Here we observed that morphological identification of *H*. *capsulatum* in culture took a relatively long period (median ± SD; 28 ± 34.2 days) due to fastidious fungal growth and late production of asexual reproduction structures (micro and macro conidia). Molecular methods, on the other hand, allow more rapid detection and identification of *H*. *capsulatum* in biological specimens. However, PCR-based diagnostic methods are not available in resource-limited laboratories, because it demands specific apparatus, including a thermocycler, electrophoresis device and UV light source. LAMP seems to represent a suitable alternative to this problem, because it is an isothermal nucleic acid amplification technique which can be performed with a simple water bath, at 60°C for 2 hours, providing a cost-effective early diagnosis and contributing to the decrease of histoplasmosis mortality rate, since patients with PDH usually die in 10–14 days [51].

Scheel *et al* (2014) have proposed a specific LAMP assay targeting the gene *Hcp100*, a 100 kDa protein-encoding gene of *H*. *capsulatum*. The LOD for this assay ranged from 10 fg/μL to 10 pg/μL of *H*. *capsulatum* DNA, achieving 67% of sensitivity in culture-proven urine specimens [30]. Both ITS LAMP and nPCR, proposed here, presented a LOD of 1 fg/μL. However, 100% reproducibility of these two assays was only achieved at 100 fg/μL. This result was expected, because the whole genome of *H*. *capsulatum* has approximately 33 fg, therefore, 10 fg of *H*. *capsulatum* DNA represents less than one genome and thus the assay sensitivity might be decreased.

Despite this low LOD, the ITS LAMP and nPCR achieved 54% and 64% of sensitivity in clinical specimens, respectively, when compared to culture. Likely, the lack or minimal amount of fungal DNA dispersed among the relatively abundant host DNA may contribute to the low sensitivity. The excellent agreement between ITS LAMP and ITS nPCR with *Hcp100* nPCR (0.91 for both assays; see Table 6), the molecular reference assay used, confirmed there was a lack or minimal amount of fungal DNA in some samples, producing false negative results in the molecular analyses.

In this study, we used the *Hcp100* nPCR as a molecular reference, due to its high specificity (PPV) [59] and because this gene has been the main target sequence used in many other studies on molecular detection of *H*. *capsulatum* [25,60,61]. The lower specificity of the LAMP assay in comparison with culture was caused by a false positive (sample HGT061) result of the subject HGT061 from group B. Although all reactions were carefully carried out in a sterile environment and analyzed in a separate room to avoid cross-contamination with previous LAMP or nPCR products, opening the reaction tubes after the LAMP has run, to perform analysis in electrophoresis or addition of Sybr Green, may create a risk of contamination. This could be the reason for the false positive result in patient HGT061. On the other hand, the patient HGT061 was treated with sulfamethoxazole + trimethoprim (SMX-TMP) 800 mg + 160 mg/day for 21 days and fluconazole 150 mg/day for 7 days, and showed a favorable outcome. Since SMX-TMP is known to be efficient for histoplasmosis treatment, as demonstrated *in vivo* [62] and *in vitro* [63], we cannot completely rule out the possibility of histoplasmosis in this subject.

The samples HGT028, HGT039, HGT068, HGT079 and HGT083 provided inconsistent results among molecular assays and culture. To investigate, first we sequenced these isolates to verify the presence of any polymorphisms in ITS region. The sequences showed no polymorphisms in the annealing region of our primers. Further, to rule out the presence of an amplification inhibitor, 2 ng of *H*. *capsulatum* DNA was added in each sample and the reactions became positive. LAMP and nPCR are robust methods that may detect minimal amounts of DNA, but the difficulty of extracting high-quality fungal DNA from biological specimens is still a challenging step, as observed by Scheel *et al* (2014) analyzing urine specimens using LAMP assay. We speculate that loss of DNA during extraction caused such inconsistent results.

In order to test these two molecular methods as direct assays for fast screening of PDH, samples spiked with *H*. *capsulatum* cells were analyzed without any previous DNA extraction or purification steps. Amplification was not inhibited when approximately 5% of heparin-containing whole blood was added in the reaction. However, EDTA-containing whole blood completely inhibited the ITS LAMP reaction by quelling magnesium ions. In fact, magnesium sulfate concentrations below 10 mM provided no amplification, while concentrations above 14 mM increased the rate of false-positives. LAMP has been applied in some studies as a direct screening assay in crude samples [64–66]. We have used whole blood in a direct assay because it is a less invasive sampling method and could provide better results since there is less DNA from the patient. Although this assay does not represent a real clinical specimen, since *H*. *capsulatum* is an intracellular pathogen, it shows us whether sample inhibitors could interrupt the reaction as well as whether the assays could be used directly on heparin-containing specimens. Hayashida and coworkers (2017) described a direct LAMP assay to detect *Plasmodium falciparum* and non-falciparum in heparin-containing whole blood samples. Curiously, the direct assay was more sensitive than conventional methods using purified blood DNA [66]. Although heparin is a well-known PCR inhibitor, a dilution of 20-fold of collected specimen in LAMP reaction is sufficient to obtain reliable results in the LAMP assay [67].

This is the first application of LAMP and nPCR using the ITS multicopy region to detect *H*. *capsulatum* in biological specimens. The ITS LAMP and the ITS nPCR were both able to detect *H*. *capsulatum* DNA with a low LOD, even when crude heparin-containing whole blood was used. Despite the low LOD achieved, these methods should not substitute fungal culture isolation as the gold standard in PDH diagnosis, since false negative results may occur due to lack of, or minimal amount of, fungal DNA in the sample, yet ITS LAMP has the potential for use in conjunction with culture for early diagnosis of PDH. Moreover, our results point to the possibility of direct pathogen detection in biological specimens for the diagnosis of PDH in HIV patients and in general histoplasmosis clinical conditions. However, more clinical specimens should be evaluated by ITS LAMP and nPCR to analyze the sensitivity in a broader range of clinical samples and for validation of the direct assay. In addition, sensitivity and specificity may be improved by optimizing DNA extraction methods, which include appropriate fungal cell wall breakdown, and adopting closed-tube strategies to evaluate LAMP results to avoid cross-contamination, such as pH-sensitive dyes or turbidimeter. Outside of clinical applications, these primers set could also be used to improve our knowledge about the epidemiology of *H*. *capsulatum* by its environmental detection.

## Supporting information

S1 Checklist Standards for Reporting of Diagnostic Accuracy (STARD) checklist. (PDF)

S1 Fig Standards for Reporting of Diagnostic Accuracy (STARD) flow chart of the 27 suspected histoplasmosis cases enrolled in the study. (PDF)

S2 Fig Annealing sites of the ITS LAMP and nPCR primer sets. Hc_F2 and Hc_F1 are different annealing regions of the Hc_FIP primer. Hc_B2 and Hc_B1 are annealing regions of the Hc_BIP primer. Gray letters represent ITS 1 and ITS 2 regions, respectively, and black letters represent 5.8S and LSU ribosomal RNA coding sequences, respectively, as showed in Fig 1.
(PDF)

S3 Fig Alignment of the ITS LAMP and nPCR primers set with ribosomal DNA from pathogenic and environmental fungal species. (PDF)

S4 Fig Agarose gel of GAPDH PCR product from biological specimens. (PDF)

S1 Table Reference strains of *H. capsulatum* from different clades used for validation of ITS LAMP and nPCR assays. (PDF)
