# Supplementary material for: Loop-mediated Isothermal Amplification and nested PCR of the Internal Transcribed Spacer (ITS) for Histoplasma capsulatum detection
Source: PLoS Negl Trop Dis. 2019 Aug 26;13(8):e0007692. doi: 10.1371/journal.pntd.0007692 (PMC6730939; doi:10.1371/journal.pntd.0007692)
Supplement: S1 Fig — (PDF) [file pntd.0007692.s002.pdf]

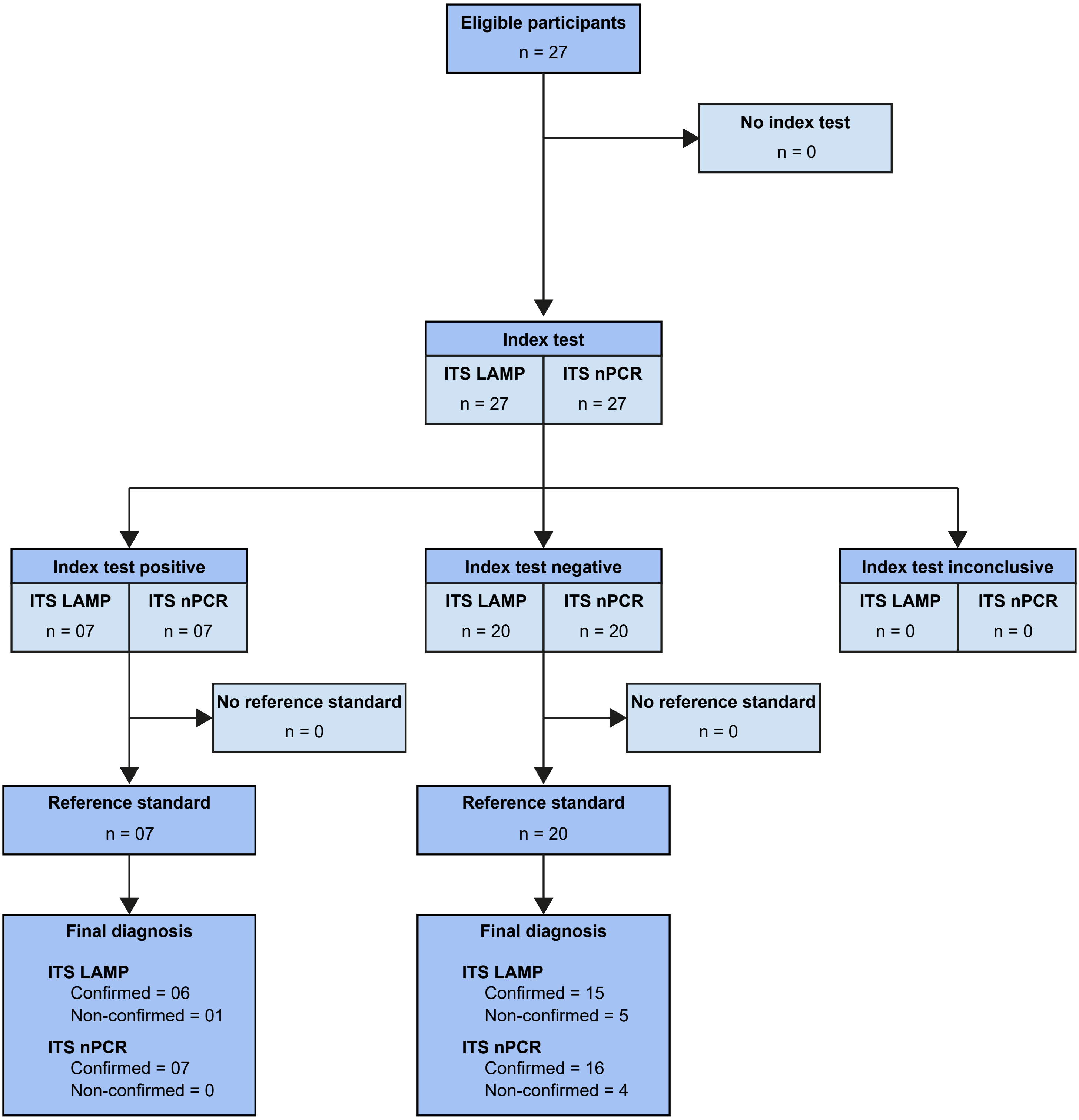
Eligible participants
n = 27
No index test
n = 0
Index test
ITS LAMP
n = 27
ITS nPCR
n = 27
Index test positive
ITS LAMP
n = 07
ITS nPCR
n = 07
Index test negative
ITS LAMP
n = 20
ITS nPCR
n = 20
Index test inconclusive
ITS LAMP
n = 0
ITS nPCR
n = 0
No reference standard
n = 0
No reference standard
n = 0
Reference standard
n = 07
Reference standard
n = 20
Final diagnosis
ITS LAMP
Confirmed = 06
Non-confirmed = 01
ITS nPCR
Confirmed = 07
Non-confirmed = 0
Final diagnosis
ITS LAMP
Confirmed = 15
Non-confirmed = 5
ITS nPCR
Confirmed = 16
Non-confirmed = 4
