# Supplementary material for: Loop-mediated Isothermal Amplification and nested PCR of the Internal Transcribed Spacer (ITS) for Histoplasma capsulatum detection
Source: PLoS Negl Trop Dis. 2019 Aug 26;13(8):e0007692. doi: 10.1371/journal.pntd.0007692 (PMC6730939; doi:10.1371/journal.pntd.0007692)
Supplement: S2 Fig — Hc_F2 and Hc_F1 are different annealing regions of the Hc_FIP primer. Hc_B2 and Hc_B1 are annealing regions of the Hc_BIP primer. Gray letters represent ITS 1 and ITS 2 regions, respectively, and black letters represent 5.8S and LSU ribosomal RNA coding sequences, respectively, as showed in Fig 1. (PDF) [file pntd.0007692.s003.pdf]

1 10 20 30 40 50 60 70

CCACGCCGTGGGGGGCTGGGAGCCTCTGACCGGGAACCCCACCGCCCCCCTACCCGGCCACCCTTGTCTA

Hc_F3

ITS_HcI

CCGGACCTGTTGCCTCGGCGGGCCTGCAGCGATGCTGCCGGGGGAGCTTCTCCTCCCCGGGCCCGTGTCC

Hc_F2 Hc_F1

ITS_HcIII

GCCGGGGACACCGCAAGAACCGTCGGTGAACGATTGGCGTCTGAGCATGAGAGCGATAATAATCCAGTC**A**

Hc_B1

AAACTTTCAACAACGGATCTCTTGGTTCCGACATCGATGAAGAACGCACCGAAATGCGATAAGTAATGTG

Hc_B2 Hc_B3

AATTGCAGAATTCCGTGAATCATCGAATCTTTGAACGCACATTGCGCCCCCTGGTATTCCGGGGGGCATG

CCTGTCCGAGCGTCATTGCAACCCTCAAGCGCGGCTTGTGTGTTGGGCCGTCGTCCCCCCTCGACCGGCG

GGACGTGCCCGAAATGCAGTGGCGGTGTCGAGTTCCGGTGCCCGAGCGTATGGGGCTTTGCCACCCGCTC

TGGAGGCCCGGCCGGCTCCGGCCCACCATGTCAACCCCCCTCTCACACCAGG**TTGACCTCGGATCAGGTA**

ITS_HcIV

GGGATACCCGCTGAACTTAAGCATATCAATGAGCGGAGGAAAAGAAACCAACAGGGATTGCCTCAGTAAC

GGCGAGTGAAGCGGCAAGAGCTCAAATTTGAAATCCGGCCCCCCTGGGGGCCTGAGTTGTAATTTGCAGA

GGATGCTTCGGGCGCGACCGCGGTCCAAGTCCCCTGGAACGGGGCGTCGTAGAGGGTGAGAATCCCGTCT

ITS_HcII

CCGGCCGGCCGGTCTCGCCCGTGTGAAGCTCCTTCGACGAGTCGAGTTGTTTGGGAATGCAGCTCCAAAT

GGGTGGTAAATTTCATCTAAAGCTAAATACTGGTCGGAGACCGATAGCGCACAAGTAGAGTGATCGAAAG

ATGAAAAGCACTTTGAAAAGAGAGTTAAACAGCATGTGAAATTGTTCAAAGGGAAGCGCTTGCGACCAGA

GTCGGCCGCGGGGGTTCAGCGGGCATTCGTTGCCCGTGCAATCCCCCGCGGCCGGGCCAGGGTCGGTTTC

GACGGCCGGTCAAAGGCCCCCGGAATGTGTCGCCTCTCGGGGCGTCTTATATCCGGGGGTGCAATGCGGC

CAGTCGGGACCGAGGAACGCGCTCCGGCACGGACGCTGGCTTAATGGTCGTCAGCGACC
