# Supplementary material for: Loop-mediated Isothermal Amplification and nested PCR of the Internal Transcribed Spacer (ITS) for Histoplasma capsulatum detection
Source: PLoS Negl Trop Dis. 2019 Aug 26;13(8):e0007692. doi: 10.1371/journal.pntd.0007692 (PMC6730939; doi:10.1371/journal.pntd.0007692)
Supplement: S3 Fig — (PDF) [file pntd.0007692.s004.pdf]

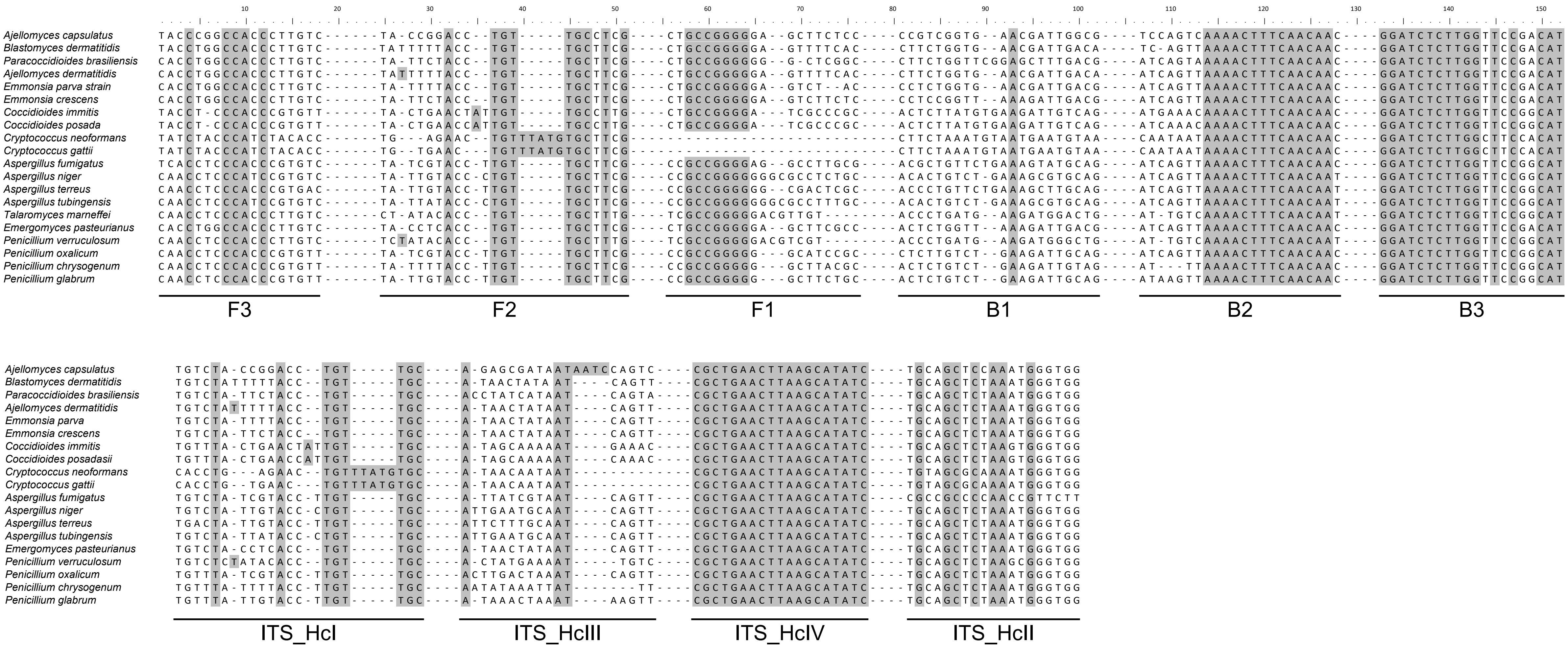

10 20 30 40 50 60 70 80 90 100 110 120 130 140 150
Ajellomyces capsulatus TACCGGCCACCCTTGTC------TA-CCGGACC--TGT-----TGCCTCG----CTGCCGGGGGA--GCTTCTCC----CCGTCGGTG--AACGATTGGCG----TCCAGTCAAAACTTTCAACAAC----GGATCTCTTGGTTCCGACAT
Blastomyces dermatitidis TACCTGGCCACCCTTGTC------TATTTTTACC--TGT-----TGCTTCG----CTGCCGGGGGA--GTTTTCAC----CTTCTGGTG--AACGATTGACA----TC-AGTTAAAACTTTCAACAAC----GGATCTCTTGGTTCCGACAT
Paracoccidioides brasiliensis CACCTGGCCACCCTTGTC------TA-TTCTACC--TGT-----TGCTTCG----CTGCCGGGGGG--G-CTCGGC----CTTCTGGTTCGGAGCTTTGACG----ATCAGTAAAAACTTTCAACAAC----GGATCTCTTGGTTCCGACAT
Ajellomyces dermatitidis CACCTGGCCACCCTTGTC------TATTTTTACC--TGT-----TGCTTCG----CTGCCGGGGGA--GTTTTCAC----CTTCTGGTG--AACGATTGACA----ATCAGTTAAAACTTTCAACAAC----GGATCTCTTGGTTCCGACAT
Emmonsia parva strain CACCTGGCCACCCTTGTC------TA-TTTTACC--TGT-----TGCTTCG----CTGCCGGGGGA--GTCT--AC----CCTCTGGTG--AACGATTGACG----ATCAGTTAAAACTTTCAACAAC----GGATCTCTTGGTTCCGACAT
Emmonsia crescens CACCTGGCCACCCTTGTC------TA-TTCTACC--TGT-----TGCTTCG----CTGCCGGGGGA--GTCTTCTC----CCTCCGGTT--AAAGATTGACG----ATCAGTTAAAACTTTCAACAAC----GGATCTCTTGGTTCCGACAT
Coccidioides immitis TACCT-CCCACCCGTGTT------TA-CTGAACTATTGT-----TGCCTTG----CTGCCGGGGA---TCGCCCGC----ACTCTTATGTGAAGATTGTCAG----ATGAAACAAAACTTTCAACAAC----GGATCTCTTGGTTCCGGCAT
Coccidioides posada TACCT-CCCACCCGTGTT------TA-CTGAACCATTGT-----TGCCTTG----CTGCCGGGGA---TCGCCCGC----ACTCTTATGTGAAGATTGTCAG----ATCAAACAAAACTTTCAACAAC----GGATCTCTTGGTTCCGGCAT
Cryptococcus neoformans TATCTACCCATCTACACC------TG---AGAAC--TGTTTATGTGCTTCG--------------------------CTTCTAAATGTAATGAATGTAA----CAATAATAAAACTTTCAACAAC----GGATCTCTTGGCTTCCACAT
Cryptococcus gattii TATCTACCCATCTACACC------TG--TGAAC---TGTTTATGTGCTTCG--------------------------CTTCTAAATGTAATGAATGTAA----CAATAATAAAACTTTCAACAAC----GGATCTCTTGGCTTCCACAT
Aspergillus fumigatus TCACCTCCCACCCGTGTC------TA-TCGTACC-TTGT-----TGCTTCG----CCGCCGGGGAG--GCCTTGCG----ACGCTGTTCTGAAAGTATGCAG----ATCAGTTAAAACTTTCAACAAC----GGATCTCTTGGTTCCGGCAT
Aspergillus niger CAACCTCCCATCCGTGTC------TA-TTGTACC-CTGT-----TGCTTCG----CCGCCGGGGGGGCCTCTGC----ACACTGTCT-GAAAGCGTGCAG----ATCAGTTAAAACTTTCAACAAT----GGATCTCTTGGTTCCGGCAT
Aspergillus terreus CAACCTCCCACCCGTGAC------TA-TTGTACC-TTGT-----TGCTTCG----CCGCCGGGGGGG-CGACTCGC----ACCCTGTTCTGAAAGCTTGCAG----ATCAGTTAAAACTTTCAACAAT----GGATCTCTTGGTTCCGGCAT
Aspergillus tubingensis CAACCTCCCATCCGTGTC------TA-TTATACC-CTGT-----TGCTTCG----CCGCCGGGGGGGCGCCTTTGC----ACACTGTCT-GAAAGCGTGCAG----ATCAGTTAAAACTTTCAACAAT----GGATCTCTTGGTTCCGGCAT
Talaromyces marneffei CAACCTCCCACCCTTGTC------CT-ATACACC--TGT-----TGCTTTG----TCGCCGGGGGACGTTGT--------ACCCTGATG--AAGATGGACTG----AT-TGTCAAAACTTTCAACAAT----GGATCTCTTGGTTCCGGCAT
Emergomyces pasteurianus CACCTGGCCACCCTTGTC------TA-CCTCACC--TGT-----TGCTTCG----CTGCCGGGGGA--GCTTCGCC----ACTCTGGTT--AAAGATTGACG----ATCAGTTAAAACTTTCAACAAC----GGATCTCTTGGTTCCGACAT
Penicillium verruculosum CAACCTCCCACCCTTGTC------TCTATACACC--TGT-----TGCTTTG----TCGCCGGGGGACGTCGT--------ACCCTGATG--AAGATGGGCTG----AT-TGTCAAAACTTTCAACAAT----GGATCTCTTGGTTCCGGCAT
Penicillium oxalicum CAACCTCCCACCCGTGTT------TA-TCGTACC-TTGT-----TGCTTCG----CCGCCGGGGG---GCATCCGC----CTCTTGTCT--GAAGATTGCAG----ATCAGTTAAAACTTTCAACAAC----GGATCTCTTGGTTCCGGCAT
Penicillium chrysogenum CAACCTCCCACCCGTGTT------TA-TTTTACC-TTGT-----TGCTTCG----CCGCCGGGGG---GCTTACGC----ACTCTGTCT--GAAGATTGTAG----AT---TTAAAACTTTCAACAAC----GGATCTCTTGGTTCCGGCAT
Penicillium glabrum CAACCTCCCACCCGTGTT------TA-TTGTACC-TTGT-----TGCTTCG----CCGCCGGGGG---GCTTCTGC----ACTCTGTCT--GAAGATTGCAG----ATAAGTTAAAACTTTCAACAAC----GGATCTCTTGGTTCCGGCAT
F3
F2
F1
B1
B2
B3
Ajellomyces capsulatus TGTCTA-CCGGACC--TGT-----TGC----A-GAGCGATAATAATCCAGTC----CGCTGAACTTAAGCATATC----TGCAGCTCCAAATGGGTGG
Blastomyces dermatitidis TGTCTATTTTTACC--TGT-----TGC----A-TAACTATAAT----CAGTT----CGCTGAACTTAAGCATATC----TGCAGCTCTAAATGGGTGG
Paracoccidioides brasiliensis TGTCTA-TTCTACC--TGT-----TGC----ACCTATCATAAT----CAGTA----CGCTGAACTTAAGCATATC----TGCAGCTCTAAATGGGTGG
Ajellomyces dermatitidis TGTCTATTTTTACC--TGT-----TGC----A-TAACTATAAT----CAGTT----CGCTGAACTTAAGCATATC----TGCAGCTCTAAATGGGTGG
Emmonsia parva TGTCTA-TTTTACC--TGT-----TGC----A-TAACTATAAT----CAGTT----CGCTGAACTTAAGCATATC----TGCAGCTCTAAATGGGTGG
Emmonsia crescens TGTCTA-TTCTACC--TGT-----TGC----A-TAACTATAAT----CAGTT----CGCTGAACTTAAGCATATC----TGCAGCTCTAAATGGGTGG
Coccidioides immitis TGTTTA-CTGAACTATTGT-----TGC----A-TAGCAAAAAT----GAAAC----CGCTGAACTTAAGCATATC----TGCAGCTCTAAGTGGGTGG
Coccidioides posadasii TGTTTA-CTGAACCATTGT-----TGC----A-TAGCAAAAAT----CAAAC----CGCTGAACTTAAGCATATC----TGCAGCTCTAAGTGGGTGG
Cryptococcus neoformans CACCTG---AGAAC--TGTTTATGTGC----A-TAACAATAAT-----------CGCTGAACTTAAGCATATC----TGTAGCGCAAAATGGGTGG
Cryptococcus gattii CACCTG--TGAAC---TGTTTATGTGC----A-TAACAATAAT-----------CGCTGAACTTAAGCATATC----TGTAGCGCAAAATGGGTGG
Aspergillus fumigatus TGTCTA-TCGTACC-TTGT-----TGC----A-TTATCGTAAT----CAGTT----CGCTGAACTTAAGCATATC----CGCCGCCCCAACCGTTCTT
Aspergillus niger TGTCTA-TTGTACC-CTGT-----TGC----ATTGAATGCAAT----CAGTT----CGCTGAACTTAAGCATATC----TGCAGCTCTAAATGGGTGG
Aspergillus terreus TGACTA-TTGTACC-TTGT-----TGC----ATTCTTTGCAAT----CAGTT----CGCTGAACTTAAGCATATC----TGCAGCTCTAAATGGGTGG
Aspergillus tubingensis TGTCTA-TTATACC-CTGT-----TGC----ATTGAATGCAAT----CAGTT----CGCTGAACTTAAGCATATC----TGCAGCTCTAAATGGGTGG
Emergomyces pasteurianus TGTCTA-CCTCACC--TGT-----TGC----A-TAACTATAAT----CAGTT----CGCTGAACTTAAGCATATC----TGCAGCTCTAAATGGGTGG
Penicillium verruculosum TGTCTCTATACACC--TGT-----TGC----A-CTATGAAAAT-----TGTC----CGCTGAACTTAAGCATATC----TGCAGCTCTAAGCGGGTGG
Penicillium oxalicum TGTTTA-TCGTACC-TTGT-----TGC----ACTTGACTAAAT----CAGTT----CGCTGAACTTAAGCATATC----TGCAGCTCTAAATGGGTGG
Penicillium chrysogenum TGTTTA-TTTTACC-TTGT-----TGC----AATATAAATTAT-------TT----CGCTGAACTTAAGCATATC----TGCAGCTCTAAATGGGTGG
Penicillium glabrum TGTTTA-TTGTACC-TTGT-----TGC----A-TAAACTAAAT----AAGTT----CGCTGAACTTAAGCATATC----TGCAGCTCTAAATGGGTGG
ITS_HcI
ITS_HcIII
ITS_HcIV
ITS_HcII
