# Supplementary figures and images for: Loop-mediated Isothermal Amplification and nested PCR of the Internal Transcribed Spacer (ITS) for Histoplasma capsulatum detection
Source: PLoS Negl Trop Dis. 2019 Aug 26;13(8):e0007692. doi: 10.1371/journal.pntd.0007692 (PMC6730939; doi:10.1371/journal.pntd.0007692)

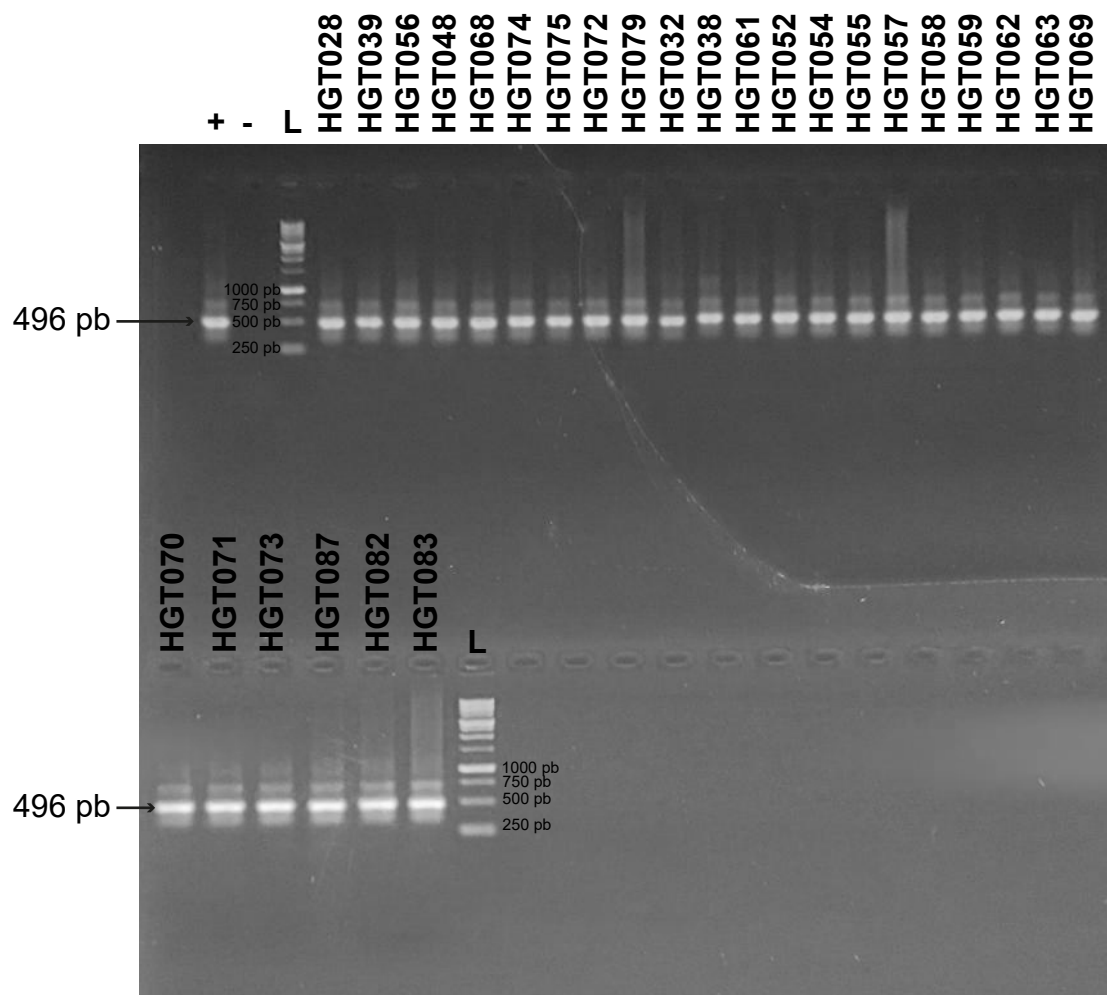

Supplement: S4 Fig — (PDF) [file pntd.0007692.s005.pdf]
