# Supplementary material for: Loop-mediated Isothermal Amplification and nested PCR of the Internal Transcribed Spacer (ITS) for Histoplasma capsulatum detection
Source: PLoS Negl Trop Dis. 2019 Aug 26;13(8):e0007692. doi: 10.1371/journal.pntd.0007692 (PMC6730939; doi:10.1371/journal.pntd.0007692)
Supplement: S1 Table — (PDF) [file pntd.0007692.s006.pdf]

S1 Table. Reference strains of *H. capsulatum* from different clades used for validation of ITS LAMP and nPCR assays.

Variety	Isolate§	Clades‡	Origin
capsulatum	2767	North America 2 (NAm2)	Alabama/EUA
capsulatum	1001	North America 1 (NAm1)	Washington/EUA
capsulatum	2358	Latin America A (LAmA)	Medellin/Colombia
capsulatum	2363	Latin America B (LAmB)	Medellin/Colombia
duboisii	2444	Africa	Guinea-Liberian Border
capsulatum	4741	Netherlands	Netherlands
capsulatum	2357	Linage H66	Medellin/Colombia
capsulatum	2360	Linage H69	Medellin/Colombia
capsulatum	2431	Linage H81	Panama

‡ According Kasuga *et al,* 2003.

§ Roche Molecular Systems Culture Collection, Alameda, CA, USA.
